# Use of Endoscopic Images in the Prediction of Submucosal Invasion of Gastric Neoplasms: Automated Deep Learning Model Development and Usability Study

**DOI:** 10.2196/25167

**Published:** 2021-04-15

**Authors:** Chang Seok Bang, Hyun Lim, Hae Min Jeong, Sung Hyeon Hwang

**Affiliations:** 1 Department of Internal Medicine Hallym University College of Medicine Chuncheon Republic of Korea; 2 Institute for Liver and Digestive Diseases Hallym University Chuncheon Republic of Korea; 3 Institute of New Frontier Research Hallym University College of Medicine Chuncheon Republic of Korea; 4 Division of Big Data and Artificial Intelligence Chuncheon Sacred Heart Hospital Chuncheon Republic of Korea

**Keywords:** convolutional neural network, deep learning, automated deep learning, endoscopy, gastric neoplasms, neural network, deep learning model, artificial intelligence

## Abstract

**Background:**

In a previous study, we examined the use of deep learning models to classify the invasion depth (mucosa-confined versus submucosa-invaded) of gastric neoplasms using endoscopic images. The external test accuracy reached 77.3%. However, model establishment is labor intense, requiring high performance. Automated deep learning (AutoDL) models, which enable fast searching of optimal neural architectures and hyperparameters without complex coding, have been developed.

**Objective:**

The objective of this study was to establish AutoDL models to classify the invasion depth of gastric neoplasms. Additionally, endoscopist–artificial intelligence interactions were explored.

**Methods:**

The same 2899 endoscopic images that were employed to establish the previous model were used. A prospective multicenter validation using 206 and 1597 novel images was conducted. The primary outcome was external test accuracy. Neuro-T, Create ML Image Classifier, and AutoML Vision were used in establishing the models. Three doctors with different levels of endoscopy expertise were asked to classify the invasion depth of gastric neoplasms for each image without AutoDL support, with faulty AutoDL support, and with best performance AutoDL support in sequence.

**Results:**

The Neuro-T–based model reached 89.3% (95% CI 85.1%-93.5%) external test accuracy. For the model establishment time, Create ML Image Classifier showed the fastest time of 13 minutes while reaching 82.0% (95% CI 76.8%-87.2%) external test accuracy. While the expert endoscopist's decisions were not influenced by AutoDL, the faulty AutoDL misled the endoscopy trainee and the general physician. However, this was corrected by the support of the best performance AutoDL model. The trainee gained the most benefit from the AutoDL support.

**Conclusions:**

AutoDL is deemed useful for the on-site establishment of customized deep learning models. An inexperienced endoscopist with at least a certain level of expertise can benefit from AutoDL support.

## Introduction

Artificial intelligence (AI) using deep learning (DL), which mimics the intellectual function of humans, has been increasingly adopted in clinical medicine, especially for cognitive function in computer vision [1-3], including automated image recognition, classification, and segmentation tasks [4-6]. Application of AI to endoscopic examination is noninvasive and can further help in detecting hidden or hard-to-detect lesions in real time. Moreover, automated determination of the optimum classification—providing delineation of the lesions—may be helpful to endoscopists, especially for inexperienced physicians. Optimizing classification facilitates the appropriate selection of high-risk patients who need additional workup or treatment [4,7]. Current established AI models are in the research-based format, which tend to have limited value in real-world clinical practice. However, AI models can potentially be used as add-on testing as a secondary assistant observer for endoscopists.

The accurate prediction of invasion depth for gastric neoplasms is an essential skill of endoscopists [8,9]. Gastric neoplasms confined to the mucosa or superficial submucosa are potential candidates for endoscopic resection [9]. Thus, precisely predicting the invasion depth is essential for determining the therapeutic strategy. Prediction of the invasion depth is based on the gross morphology of the lesions, and there are no standard criteria for classifying invasion depth. Therefore, current practice is limited by the inevitable interobserver variability and inaccurate determination of the invasion depth in gastric neoplasms [9].

The authors previously established DL models for classifying the invasion depth (mucosa-confined versus submucosa-invaded) of gastric neoplasms from endoscopic images using transfer learning of pretrained convolutional neural networks (CNNs) based on the PyTorch platform [10]. The external test accuracy was able to reach 77.3% [9]. However, the establishment of a DL model requires substantial time, and high performance is needed before applying these models to real-world clinical practice.

Automated deep learning (AutoDL) techniques, which enable fast searching of optimal neural architectures and hyperparameters without complex coding, have been widely developed. This “off-the-shelf” software or platform can be used without professional AI expertise and can easily be applied to clinical practice with simple inference structures [11]. However, the performance of AI models established by data scientists in the traditional manner and AutoDL models established by health care researchers for the gastrointestinal endoscopy field have not been directly compared. Moreover, there are scarce data in terms of human-AI interactions. For example, the reaction of endoscopists (ie, approval, indolence, or disregard) to diagnoses made using an AI model remains unknown [12]. This study aimed to establish AutoDL models classifying invasion depth of gastric neoplasms using endoscopic images and compare the diagnostic performance of the AutoDL models with previous CNN models established in the traditional way. Additionally, endoscopist-AI interactions using the newly established model were further examined.

## Methods

### Construction of the Data Set

**Figure 1:**
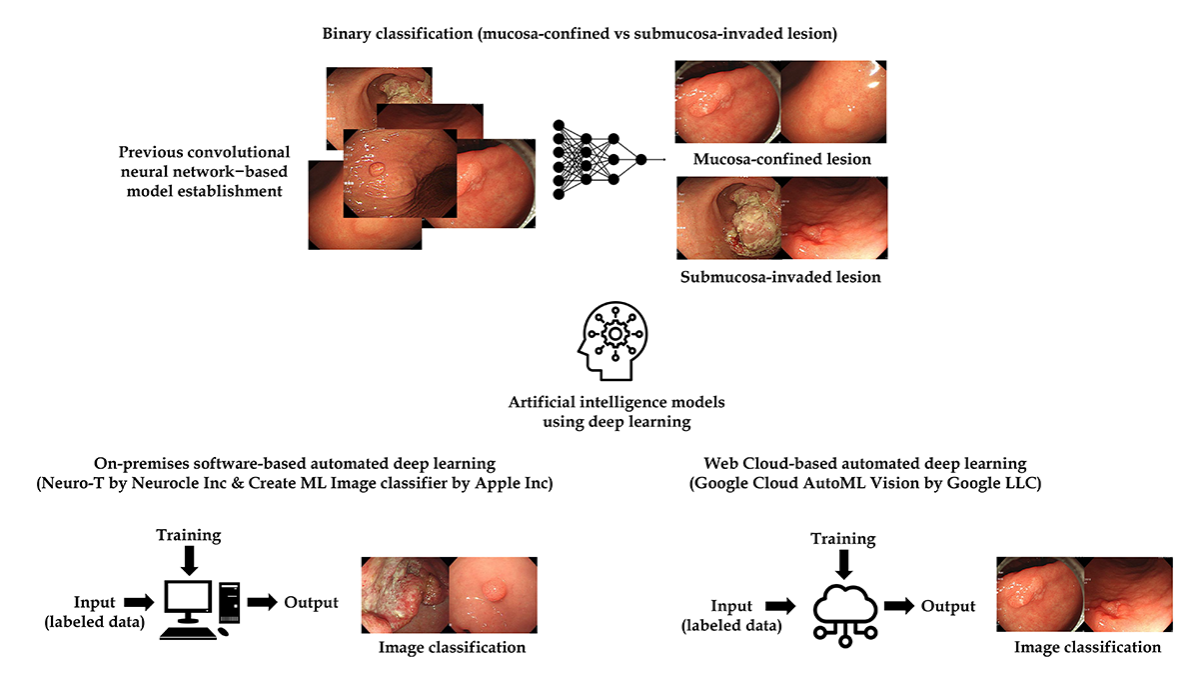
Schematic flow for the establishment of automated deep learning models in data construction.

**Figure 2:**
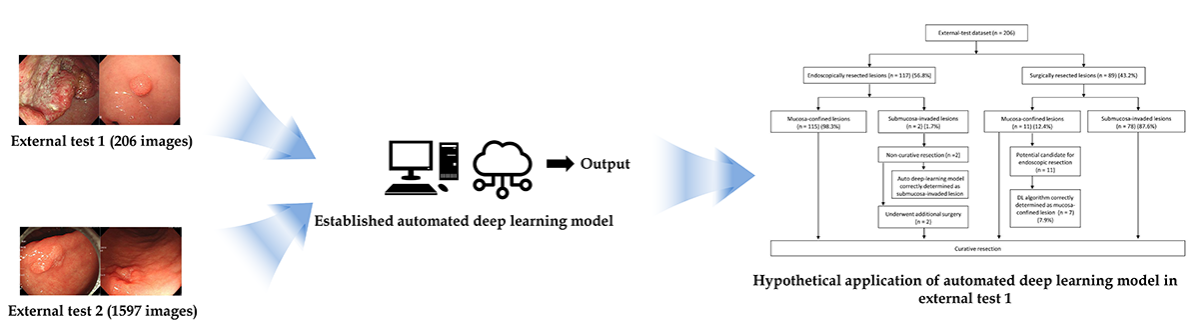
Schematic flow for the establishment of automated deep learning models in performance evaluation.

This study extends the previous research on this topic [9] by constructing (Figure 1) and evaluating (Figure 2) experimental DL models with AutoDL tools. In order to compare the diagnostic performance of AutoDL–based models to the previous CNN models, the same input images (2899 white-light imaging endoscopic images) that were used to establish the previous model were used again. The detailed data collection process was described previously [9]. Briefly, between 2010 and 2017 in the Chuncheon Sacred Heart Hospital (Republic of Korea), we enrolled consecutive patients with any type of gastric neoplasms discovered during upper gastrointestinal endoscopy and histologically confirmed. Endoscopic images were collected from the in-hospital database in JPEG format, with a minimum resolution of 640×480 pixels [9]. The same previously used external test data set (206 white-light imaging endoscopic images) was also used in classifying the performance of the AutoDL models. This external test data set was constructed by collecting images from consecutive patients who underwent upper gastrointestinal endoscopy between 2019 and 2020, and all the images were mutually exclusive from those of the training and internal validation data set (Table 1) [9].

To guarantee the generalizability of the performance of the newly developed AutoDL model, an additional performance verification (prospective validation) test with another external test data set was conducted. This second external test data set, including 1597 images, was collected from consecutive patients who underwent upper gastrointestinal endoscopy at the Hallym University Sacred Heart Hospital from 2018 to 2020 (Table 1).

**Table 1:** Summary of images in each data set.

		Number of images		
Invasion depth of gastric neoplasms		Training and internal validation data set	External test data set 1	External test data set 2
Overall		2899	206	1597
**Mucosa-confined lesions**		1900	126	1344
	Low-grade dysplasia	727	68	734
	High-grade dysplasia	421	21	110
	Early gastric cancer	752	37	500
**Submucosa-invaded lesions**		999	80	253
	Early gastric cancer	282	23	155
	Advanced gastric cancer	717	57	98

### AutoDL Tools Used in the Study

AutoDL tools including Neuro-T (version 2.0.2; Neurocle Inc), Create ML Image Classifier (Apple Inc), and Google Cloud AutoML Vision (Google LLC) were used in this study.

Neuro-T has been defined as an AutoDL software that can establish DL algorithms on its own for image recognition and classification based on a graphical user interface (GUI). The software’s algorithm analyzes the features of the data set and self-discovers optimal hyperparameters, thus making it easy for non-AI experts to build the best models. Neuro-T also offers a platform to establish anomaly detection models (supervised anomaly detection based on the clustering algorithm with deep neural networks). Anomaly detection is the identification of observations that raise suspicions by differing significantly from the majority of the training data. The neural network clustering algorithm clusters each training sample and makes its own cluster decision boundary for classifying the test sample into normal class or abnormal class. Meanwhile, Create ML is defined as a framework used to establish customized DL models on the Mac operating system (Apple Inc); Image Classifier can be accessed by GUI or Swift language code. DL models can be established using image data sets through the self-learning process of specific features. Google Cloud AutoML Vision is a web-based service to build customized DL models with automatic neural architecture searching and feature extraction. These 3 AutoDL tools were used based on the manner of the GUI (ie, no coding tool required) to build the DL models.

### Preprocessing of Images

The authors used data augmentation methods—such as rotation, and horizontal or vertical flipping of included images—and image normalization with linear transformation in terms of 3 RGB channels in order to build the previous DL model [9]. However, AutoDL tools are determined to have data preprocessing functions. Neuro-T has an automated image normalization process with a resizing function for input images. All of the included images were resized with a resolution of 512×480 pixels while building the Neuro-T–based models. Create ML Image Classifier offers GUI–based data augmentation options. These include 6 image data augmentation methods, such as “add noise,” “blur,” “crop,” “expose,” “flip,” or “rotate” functions. In order to identify the best models, we conducted multiple experiments (with or without data augmentation and single or combination data augmentation options) in Create ML. In terms of AutoML Vision, no GUI-based data augmentation option was determined. Developers can add image augmentation codes using a Python application programming interface. However, considering that the aim of this study was to develop AutoDL models without complex coding or AI expertise, we only selected the GUI-based function without data augmentation while building the AutoML Vision–based models.

### Training of AutoDL Models

The 2899 input images were uploaded to each AutoDL tool. Neuro-T and Create ML were considered on-premise software; however, AutoML Vision is a cloud-based service. The input images were uploaded to Neuro-T and Create ML, and a bucket in Google Cloud Storage system was used for data uploading in the AutoML Vision. After selecting data preprocessing options (as described above, including resizing/normalization in Neuro-T and image augmentation in Create ML), AutoDL models were trained in each specified way of self-learning.

Images were then randomly split into training and internal validation sets. The Neuro-T variable options—such as 9:1, 8:2, or 7:3—were set as per the user’s preference. Multiple experiments were further conducted to determine the model with the best performance with variable splitting ratios. However, Create ML Image Classifier automatically sets an internal validation set using approximately 5.1% of the images; thus, 149 images were allocated in the internal validation data set. In AutoML Vision, the ratio of training, internal validation, and internal test sets was 8:1:1. For the training of the anomaly detection model in Neuro-T, only images with mucosa-confined lesions could be used. Therefore, 1714 mucosa-confined images were used for training, and 186 mucosa-confined images and 999 submucosa-invaded images were used for the internal validation set. The number of iterations in training can be set for Create ML. Experiments for the different iteration numbers were conducted to prevent overfitting (ie, model learns too much about training images, and predictions are not well generalized to new images) [3].

The hardware system used for the training included NVIDIA GeForce RTX 2080 Ti graphics processing units (GPUs), dual Intel Xeon central processing units (CPUs), and 256 GB RAM for the Neuro-T–based AutoDL models. Create ML–based models were established on both the MacBook Pro laptop (2019 version, AMD Radeon Pro 5500M GPU, Intel Core i9 CPU, and 32 GB RAM) and the Mac Pro workstation (2019 version, AMD Radeon Pro Vega II GPU, Intel Xeon W CPU, and 192 GB RAM) environments in order to compare the training time according to the hardware system.

### Endoscopist-AI Interactions

**Figure 3:**
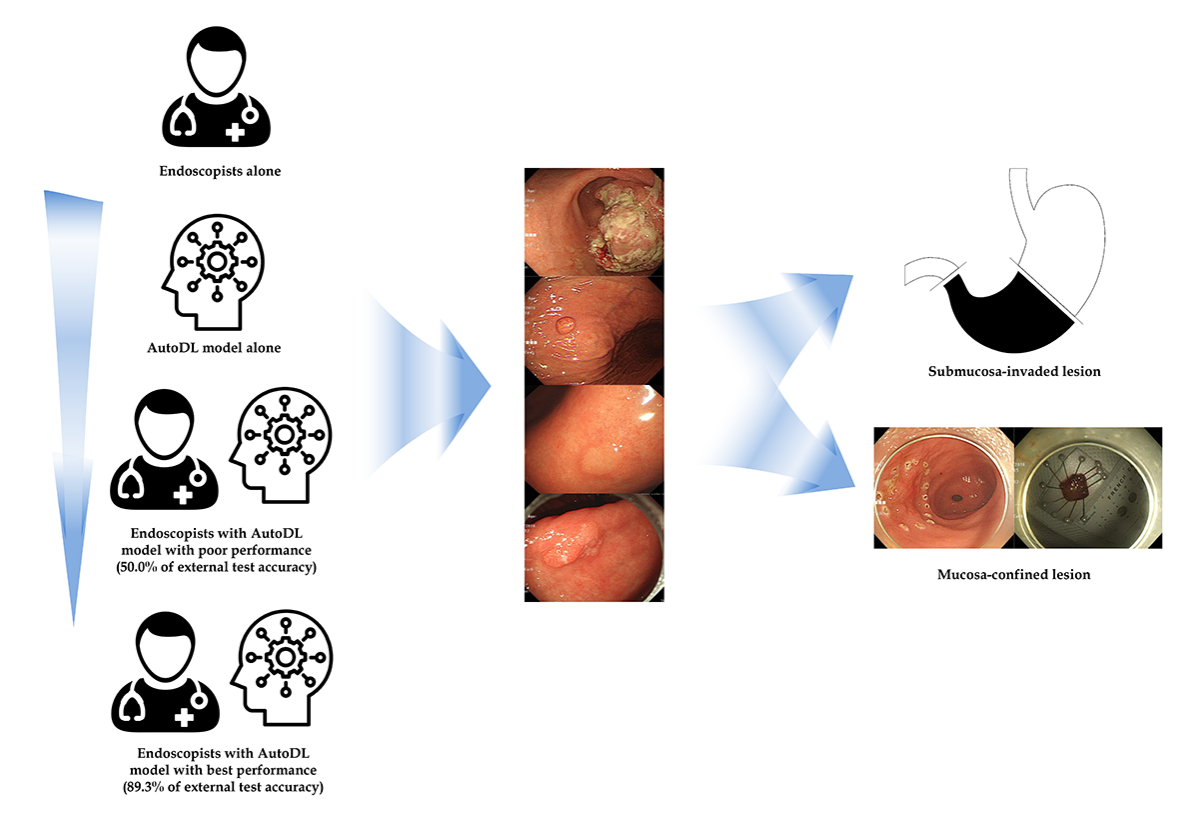
Schematic flow for the establishment of automated deep learning (AutoDL) models in endoscopist–artificial intelligence interaction test.

Three doctors with different levels of endoscopy expertise were asked to classify the invasion depth of gastric neoplasms for each endoscopic image without AutoDL support, with faulty AutoDL support, and with the support of the best performance AutoDL in sequence (Figure 3). A board-certified endoscopist with more than 7 years of endoscopic submucosal dissection experience, an endoscopy trainee, and a general physician with minimal endoscopy expertise participated in the blind test. Endoscopic images (206 images from the external test data set) without information about invasion depth were used. The first test was conducted without AI support. To find the interaction between endoscopists and poor-quality AI, blind testing was conducted while providing the poor-quality model’s (faulty AI) answers with only 50.0% external test accuracy. Another round of blind testing was performed while providing the answers from the best performance AutoDL model (89.3% external test accuracy). The confidence of the raters in their answers was recorded for each test.

A detailed description of the primary outcome and statistics are described in Multimedia Appendix 1.

## Results

### Characteristics of the Included Images

The detailed characteristics of the images included in this study were described in the previous report [9]. In brief, 65.5% of the images were mucosa-confined lesions and 34.5% were determined to be submucosa-invaded lesions (2899 images for the training and internal validation set). For the first external test (206 images), 61.2% and 38.8% of the images were determined to be mucosa-confined lesions and submucosa-invaded lesions, respectively. For the second external test (1597 images), 84.2% and 15.8% were identified to be mucosa-confined lesions and submucosa-invaded lesions, respectively. Table 1 shows a summary of the images used in this study.

### Diagnostic Performance of AutoDL Models for the First External Test

The Neuro-T–based classification model reached 89.3% (95% CI 85.1%-93.5%) accuracy, 89.1% (95% CI 84.8%-93.4%) average precision, 88.4% (95% CI 84.0%-92.8%) average recall, and 88.7% (95% CI 84.4%-93.0%) F1 score in the external test. The total training time was approximately 13 hours. The external test accuracy of the Neuro-T–based model was significantly higher than that of the previous CNN model with the best performance (ie, 77.3%, 95% CI 75.4%-79.3%; *P*=.005). The confusion matrix for the Neuro-T–based model in the external test is illustrated in Multimedia Appendix 2A. The detailed information of the established model is as follows: batch size, 80; epochs, 84; number of layers, 53; optimizer, Adam; and input height and width, 480×512 pixels. All images were resized with interlinear interpolation, and the initial learning rate was 0.002.

The anomaly detection model established by Neuro-T was able to reach 49.5% (95% CI 42.7%-56.3%) accuracy, 51.0% (95% CI 44.2%-57.8%) average precision, 51.0% (95% CI 44.2%-57.8%) average recall, and 51.0% (95% CI 44.2%-57.8%) F1 score in the external test. The training time was approximately 20 minutes. The confusion matrix for the anomaly detection model is illustrated in Multimedia Appendix 2B.

For the Create ML Image Classifier, data augmentation options combining “blur” and “rotate” provided the best performance after 25 iterations. The external test accuracy reached 83.5% (95% CI 78.4%-88.6%). The training time was approximately 76 minutes in the laptop environment, which was determined to be not significantly different from the training time in the workstation environment. Furthermore, the external test accuracy of the Create ML–based model was not statistically different from that of the previous model (*P*=.26). The confusion matrix for the Create ML–based AutoDL model with the best performance is presented in Multimedia Appendix 2C.

The fastest model establishment with high performance was achieved by data augmentation options combining “add noise” and “blur” after 25 iterations. The external test accuracy reached 82.0% (95% CI 76.8%-87.2%). The training time was determined to be only about 13 minutes in the laptop environment, which was not different from the training time in the workstation environment. Also, the external test accuracy of the Create ML–based model was not statistically different from that of the previously established CNN model (*P*=.45). The confusion matrix for the Create ML–based AutoDL model with the fastest building time and high performance is illustrated in Multimedia Appendix 2D.

For the Google Cloud AutoML Vision model, external test accuracy reached 83.0% (95% CI 77.9%-88.1%). The training time was only 25 minutes (web cloud–based environment). The external test accuracy for the AutoML Vision–based model was not statistically different from that of the previous CNN model (*P*=.31). The confusion matrix for the AutoML Vision–based model is illustrated in Multimedia Appendix 2E.

The summary statistics of external test accuracy with internal validation accuracy are shown in Table 2.

**Table 2:** Summary of external test accuracy with internal validation accuracy for each automated deep learning (AutoDL) model.

AutoDL model		Accuracy, % (95% CI)	Precision, % (95% CI)	Recall, % (95% CI)	F1 score, % (95% CI)	Training time (minutes)
**Neuro-T–based model**						826
	Internal validation performance (n=290)	92.4 (89.3-95.5)	M^a^: 92.0 (88.1-95.9); SM^b^: 93.3 (88.4-98.2)	M: 96.8 (94.3-99.3); SM: 84 (76.8-91.2)	M: 94.4 (91.1-97.7); SM: 88.4 (82.1-94.7)	
	External test performance (n=290)	89.3 (85.1-93.5)	M: 89.9 (84.6-95.2); SM: 88.3 (81.3-95.3)	M: 92.8 (88.3-97.3); SM: 84.0 (76.0-92.0)	M: 91.3 (86.4-96.2); SM: 86.1 (78.6-93.6)	
**Neuro-T–based anomaly detection model**						20
	Internal validation performance (n=1185)	80.2 (77.9-82.5)	M: 33.3 (26.6-40.0); SM: 86.2 (84.1-88.3)	M: 23.7 (17.7-29.7); SM: 91.0 (89.2-92.8)	M: 27.7 (21.3-34.1); SM: 88.6 (86.6-90.6)	
	External test performance (n=206)	49.5 (42.7-56.3)	M: 61.8 (53.3-70.3); SM: 40.2 (29.5-50.9)	M: 44.0 (35.3-52.7); SM: 58.0 (47.3-68.7)	M: 51.4 (42.6-60.2); SM: 47.5 (36.6-58.4)	
**Create ML–based model 1**						76
	Internal validation performance (n=149)	81.9 (75.7-88.1)	M: 80.6 (72.2-89.0); SM: 83.9 (75.0-92.8)	M: 89.3 (82.7-95.9); SM: 72.3 (61.4-83.2)	M: 84.7 (77.0-92.4); SM: 77.7 (67.6-87.8)	
	External test performance (n=206)	83.5 (78.4-88.6)	M: 82.3 (75.6-89.0); SM: 86.2 (78.7-93.7)	M: 92.8 (88.3-97.3); SM: 69.1 (59.0-79.2)	M: 87.2 (81.3-93.1); SM: 76.7 (67.5-85.9)	
**Create ML–based model 2**						13
	Internal validation performance (n=149)	81.9 (75.7-88.1)	M: 82.0 (73.8-90.2); SM: 81.7 (72.3-91.1)	M: 86.9 (79.7-94.1); SM: 75.4 (64.9-85.9)	M: 84.4 (76.6-92.2); SM: 78.4 (68.4-88.4)	
	External test performance (n=206)	82.0 (76.8-87.2)	M: 79.7 (72.6-86.8); SM: 87.9 (80.8-95.0)	M: 94.4 (90.4-98.4); SM: 63.0 (52.5-73.5)	M: 86.1 (80.0-92.2); SM: 73.4 (63.8-83.0)	
**AutoML Vision–based model**						25
	Internal validation performance (n=295)	84.7 (80.6-88.8)	M: 87.0 (82.3-91.7); SM: 80 (72.2-87.8)	M: 90.2 (86.0-94.4); SM: 74.5 (66.0-83.0)	M: 88.6 (84.0-93.1); SM: 77.2 (69.1-85.3)	
	External test performance (n=206)	83.0 (77.9-88.1)	M: 80.0 (73.0-87.0); SM: 91.1 (84.9-97.3)	M: 96.0 (92.6-99.4); SM: 63.0 (52.9-73.1)	M: 87.3 (81.5-93.1); SM: 74.5 (65.0-84.0)	

^a^M: mucosa-confined lesions.

^b^SM: submucosa-invaded lesions.

### Additional Performance Verification to Gain Generalization Potential in the Second External Test

For the 1597 images in the second external test, the AutoDL model was determined to perform the best (Neuro-T–based), reaching 88.6% (95% CI 87.0%-90.2%) accuracy, 83.7% (95% CI 81.9%-85.5%) average precision, 68.5% (95% CI 66.2%-70.8%) average recall, and 75.4% (95% CI 73.3%-77.5%) F1 score.

### Hypothetical Application of AutoDL Model

Hypothetical clinical application of the established AutoDL model with the best performance was conducted using the first external test data set, assuming that the AutoDL was applied in order to determine the treatment (ie, endoscopic resection or surgical resection), based on the invasion depth of the lesion. Among the lesions with endoscopic resection (n=117), 2 lesions (1.7%) were found to invade the submucosa and were resected with additional surgery after endoscopic resection. The AutoDL model correctly determined that these were submucosa-invaded lesions. Thus, the model has the potential to prevent unnecessary endoscopic procedures. Among the lesions with surgical resection (n=89), 11 lesions (12.4%) were identified to be mucosa-confined, having the potential for endoscopic resection. The AutoDL model correctly determined the mucosa-confined lesions in 7 of the 11 patients (7.9%). Thus, the model has the potential to prevent unnecessary surgeries (Multimedia Appendix 3).

### Endoscopist-AI Interactions

**Figure 4:**
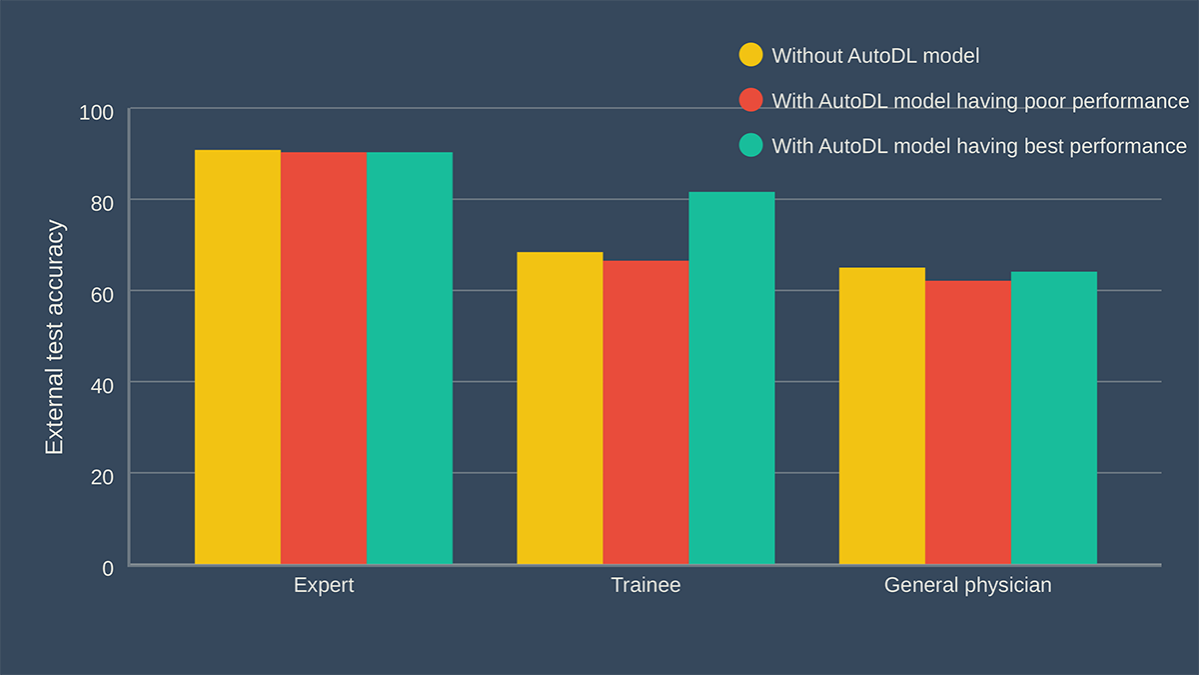
Endoscopist–artificial intelligence interactions. AutoDL: automated deep learning.

Figure 4 and Table 3 show the external test accuracy of the 3 raters. The expert endoscopist’s decision was determined to have not been influenced by the support of the AutoDL in the consecutive tests. The faulty AutoDL model misled the decisions of the endoscopy trainee and general physician, but the difference was not statistically significant. However, support from the best performance AutoDL model corrected this misdirection. The endoscopy trainee benefited the most from the support of the AutoDL model (*P*=.002). In the analysis of whether the raters were sure of their answers, confident answers showed a similar pattern to that of the overall results. In the “unconfident answers” subgroup, the expert endoscopist’s decisions were not influenced, and the trainee gained a statistically significant benefit, even with the support of the poor performance AutoDL model (*P*<.05). However, the general physician did not benefit from the support of the AutoDL models.

**Table 3:** Summary of external test accuracy in endoscopist–artificial intelligence (AI) interactions.

		External test accuracy, % (95% CI)				*P* values	
Rater		First test: endoscopist alone	Second test: endoscopist with faulty AI	Third test: endoscopist with best performance AI	First test vs second test		First test vs third test
**Expert endoscopist**							
	All answers	90.8 (86.8-94.8), (187/206)	90.3 (86.3-94.3), (186/206)	90.3 (86.3-94.3), (186/206)	.87		.87
	Confident answer	93.8 (91.7-95.9), (181/193)	90.6 (86.6-94.6), (182/201)	90.6 (86.6-94.6), (184/203)	.23		.24
	Unconfident answer	46.2 (19.1-73.3), (6/13)	80.0 (44.9-99.9), (4/5)	66.7 (13.4-99.9), (2/3)	.20		.52
**Endoscopy trainee**							
	All answers	68.4 (62.1-74.7), (141/206)	66.5 (60.1-72.9), (137/206)	81.6 (76.3-86.9), (168/206)	.67		.002
	Confident answer	80.6 (74.4-86.8), (125/155)	71.1 (63.2-79.0), (91/128)	92.2 (87.8-96.6), (130/141)	.06		.004
	Unconfident answer	31.4 (18.7-44.1), (16/51)	59.0 (48.1-69.9), (46/78)	58.5 (46.5-70.5), (38/65)	.002		.004
**General physician**							
	All answers	65.0 (58.5-71.5), (134/206)	62.1 (55.5-68.7), (128/206)	64.1 (57.5-70.7), (132/206)	.38		.84
	Confident answer	77.4 (70.0-84.8), (96/124)	71.6 (64.5-78.7), (111/155)	74.2 (67.5-80.9), (121/163)	.27		.53
	Unconfident answer	46.3 (35.5-57.1), (38/82)	33.3 (20.4-46.2), (17/51)	25.6 (12.6-38.6), (11/43)	.14		.02

### Attention Map for Explainability

**Figure 5:**
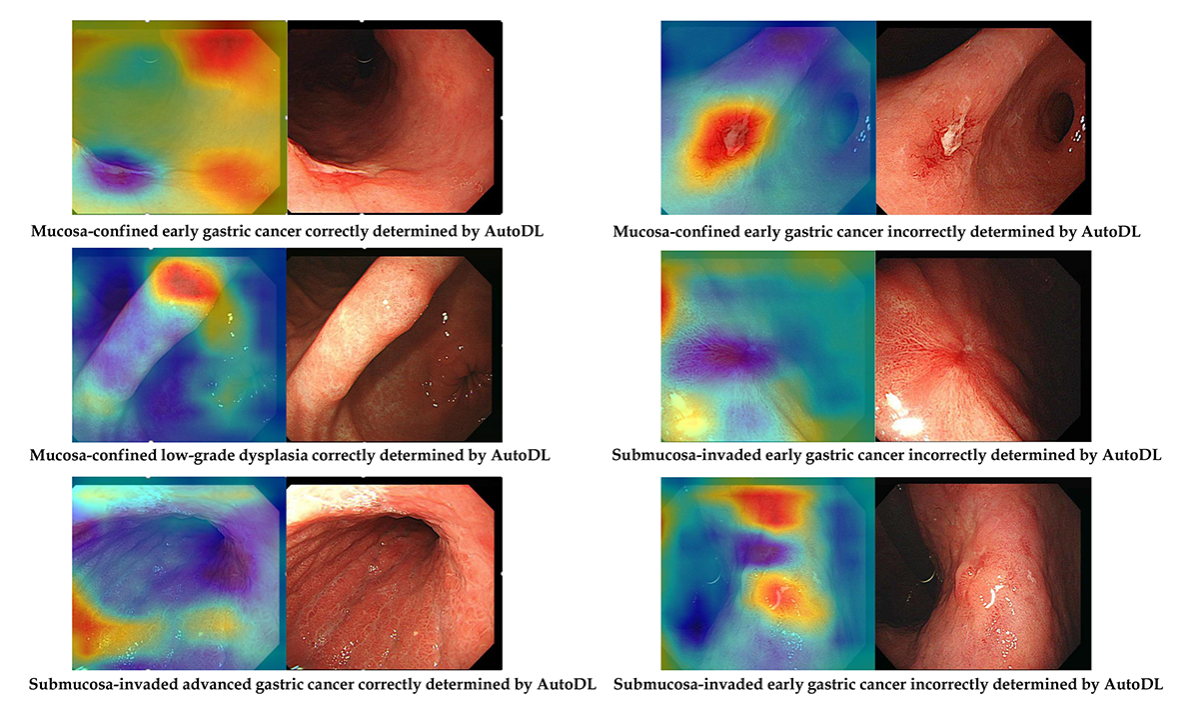
Representative samples of the attention map (Neuro-T–based model). AutoDL: automated deep learning.

A class activation map to localize the discriminative regions used by the AutoDL model to determine a specific class in the image is presented for the Neuro-T model. Figure 5 shows the correctly and incorrectly determined samples in the external test using the Neuro-T–based AutoDL model.

## Discussion

AutoDL models were established using only GUI-based systems, which surpassed or had a similar accuracy to that of the previous model for the determination of the invasion depth of gastric neoplasms using endoscopic images. The best performance model showed an external test accuracy of 89.3%, which is deemed much higher than the previous CNN model built in the traditional manner. Furthermore, the authors performed an external test to determine the generalizability, and the external test was found to exhibit robust performance. As far as the authors know, this is the highest performance achieved by an AutoDL model in the context of gastric neoplasms. In previous studies, the internal validation accuracy was 64.7% to 94.5% for the discrimination of invasion depth in gastric cancers [13-16]. However, these models can only be applied after a definite diagnosis of gastric cancer, which has limited value due to various types of gastric neoplasms in real-world clinical practice [9]. Moreover, no single study evaluated the external test accuracy; thus, the previous tests lacked generalizability.

Creating a classification model using medical images through transfer learning based on high performance CNNs is a representative establishment method for AI models. However, health care researchers often lack the AI expertise to directly apply the models to clinical data to create the AI model [17]. Data scientists and endoscopists can collaborate to create AI models. However, this process usually takes a substantial amount of time and does not immediately reflect the unmet needs of clinical practice.

AutoDL technology makes it possible for nonexperts to create high-quality DL models even without AI expertise. These tools are easy-to-use and only require data uploading to the on-premise software or web cloud platform and simple labeling for the correct classification [18]. After self-learning and model fitting, AutoDL tools provide models ready for direct inference or deployment in real-world practice. Establishing a model requires considerably less time than traditional platform-based model generation. In this study, the fastest establishment time was only 13 minutes and provided an external test accuracy (82.0%) that was similar to that of the previous CNN models based on the laptop environment. In addition, Apple’s Create ML and Google Cloud AutoML Vision are publicly available platforms that anyone can download from the App Store or access from the web cloud platform.

Endoscopists produce an enormous amount of image data in their daily practice; often, they are forced to make instantaneous medical judgments even during endoscopic procedures. However, the burnout phenomenon of endoscopists is a serious concern that needs to be addressed, as it affects concentration and, possibly, medical judgment [19]. A previous study on human-AI interactions suggested that applying a high-quality DL model to clinical decision making improves diagnostic performance compared with either DL models or physicians alone; thus, it was deemed particularly beneficial for less-experienced doctors [7].

Although AI is potentially efficacious in clinical practice, data regarding endoscopist-AI interactions remain to be scarce. Based on the findings in this study, inexperienced endoscopists with at least a certain level of expertise can benefit from AutoDL support. The endoscopy trainee—but not the general physician or expert endoscopist—benefited from AI support in this study. Most of the answers by the expert endoscopist were rated as confident and were not influenced by the AI support (ie, possible disagreement with or disregard for the AI answers). The proportion of unconfident answers in the first test was the highest for the general physician, and this proportion was markedly decreased with the support of the faulty AI in the second test. In the third test, the general physician appeared to be confused by changes in the AI answers (from the faulty AI answers to the best performance AI answers). The sequential support by the faulty AI and then the best performance AI confused the general physicians because of their minimal endoscopy expertise. This highlights not only the importance of robust answers provided by the AI but also the importance of the baseline level of experience of AI users. Therefore, the conclusion from the previous study [6] that inexperienced doctors would benefit the most from AI support was not reproduced in this study. Rather, endoscopists having at least a certain level of expertise benefited from AI support.

Although this study established a high performance AutoDL model and rigorously validated the model's performance, this analysis has several inevitable limitations originating from potential bias in data sets. First, the training images were retrieved from a single institution, which might infer a selection or spectrum bias. Because of the unique characteristics of patients in each institution, medical AI models developed from a single institution usually have limitations for widespread implementation, indicating the importance of the external test [5]. To compensate for this pitfall, we performed two rounds of prospective validations and included images from another institution. Second, the efficacy of inference for each established model was not measured in clinical practice. Each established AutoDL model employs a specified inference method, such as website-based inference or edge computing–based application inference. The efficacy of inference includes inference speed, accuracy, easy applicability, simple control flow, energy efficiency, and model size. Because inference is a different field from that of this study, another comparative study will have to be conducted for the best inference AutoDL model. Recently developed machine learning or DL models in gastrointestinal endoscopy are focused on improving the effectiveness rather than the interpretability or efficiency. The most accurate model in our study also showed longer establishment time than the other models. There have been efficiency-effectiveness trade-offs in the field of DL models. Although real-world clinical application or inference time was not the primary outcome in this study, efficiency-effectiveness trade-offs should be considered in the context of real-world settings. Third, a relatively small number of raters were included in the endoscopist-AI interaction test. We only included one representative physician in each endoscopy expertise level. Large-scale studies evaluating more discrete expertise levels would elucidate the future perspectives for the implementation of AutoDL models in the clinical setting.

AutoDL has been considered as a useful tool for the on-site establishment of customized DL models, and anyone can create an AI model with the help of AutoDL. An inexperienced endoscopist with at least a certain level of expertise can benefit from AutoDL support.

## Appendix

Multimedia Appendix 1 Primary outcome and statistics.

Multimedia Appendix 2 Confusion matrices for the automated deep learning models in the external test. (A) Neuro-T–based model. (B) Neuro-T–based anomaly detection model. (C) Create ML–based model with the best performance. (D) Create ML–based model with the fastest building time and high performance. (E) AutoML Vision–based model.

Multimedia Appendix 3 Hypothetical clinical application of the automated deep learning model for the determination of treatments based on the invasion depth of the lesion in an external test.

## Abbreviations

AI: artificial intelligence
AutoDL: automated deep learning
CNN: convolutional neural network
CPU: central processing unit
DL: deep learning
GPU: graphics processing unit
GUI: graphical user interface
