# Supplementary material for: Use of Endoscopic Images in the Prediction of Submucosal Invasion of Gastric Neoplasms: Automated Deep Learning Model Development and Usability Study
Source: J Med Internet Res. 2021 Apr 15;23(4):e25167. doi: 10.2196/25167 (PMC8085753; doi:10.2196/25167)
Supplement: Multimedia Appendix 1 [file jmir_v23i4e25167_app1.docx]

***Primary outcome and statistics***

The primary outcome was the accuracy of the established AutoDL models in discriminating invasion depth of the lesions in the external-test. Thus, the main metric was the diagnostic accuracy. The precision, recall, and F1 score calculated by each AutoDL tool in the external-test are presented. Fisher’s exact test was conducted to compare the diagnostic accuracy between AutoDL models and traditionally established CNN models and between blind-tests in the endoscopist-AI interaction experiment. A *P*-value of <0.05 was set as the statistically significant margin. The secondary outcome was the training time for each AutoDL model. All analyses were performed using R software version 3.6.3 (The R Foundation for Statistical Computing, Vienna, Austria) and Python (Python Software Foundation, version 3.7.5), with the Scikit-learn package version 0.20.3. The overall schematic flow of this study has been illustrated in Figure 1. This study was approved by the Institutional Review Board of Chuncheon Sacred Heart Hospital (number: 2018-07-003-002) and was conducted in accordance with the Declaration of Helsinki.
