# Supplementary material for: Use of Endoscopic Images in the Prediction of Submucosal Invasion of Gastric Neoplasms: Automated Deep Learning Model Development and Usability Study
Source: J Med Internet Res. 2021 Apr 15;23(4):e25167. doi: 10.2196/25167 (PMC8085753; doi:10.2196/25167)

**Multimedia Appendix 2** Confusion matrices for the automated deep-learning models in the external-test. (A) Neuro-T-based model (B) Neuro-T-based anomaly detection model (C) Create ML-based model with the best performance (D) Create ML-based model with the fastest building time and high performance (E) AutoML-Vision-based model

(A)


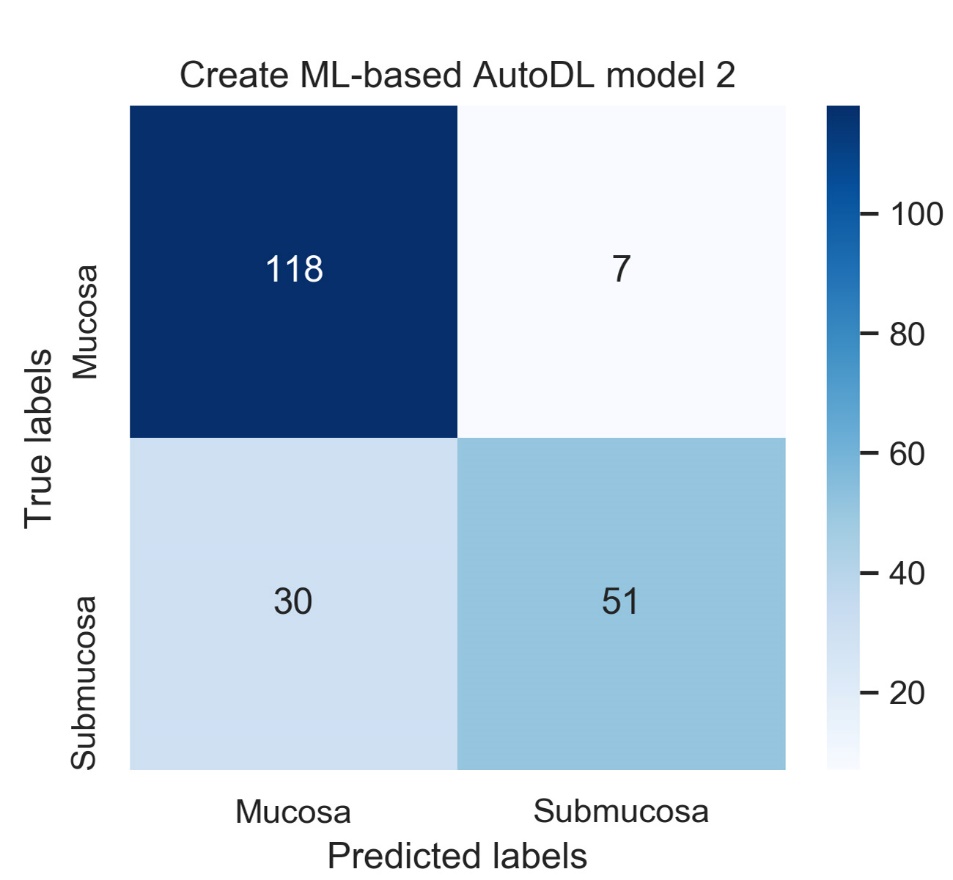


(B)


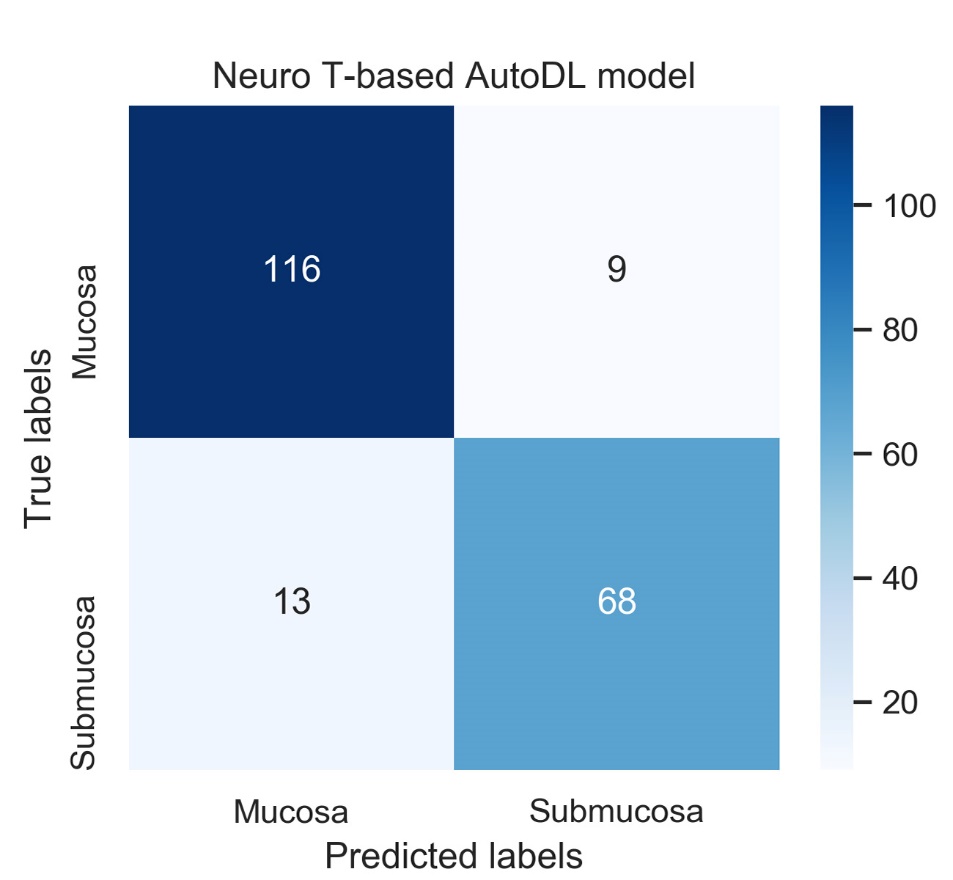


(C)


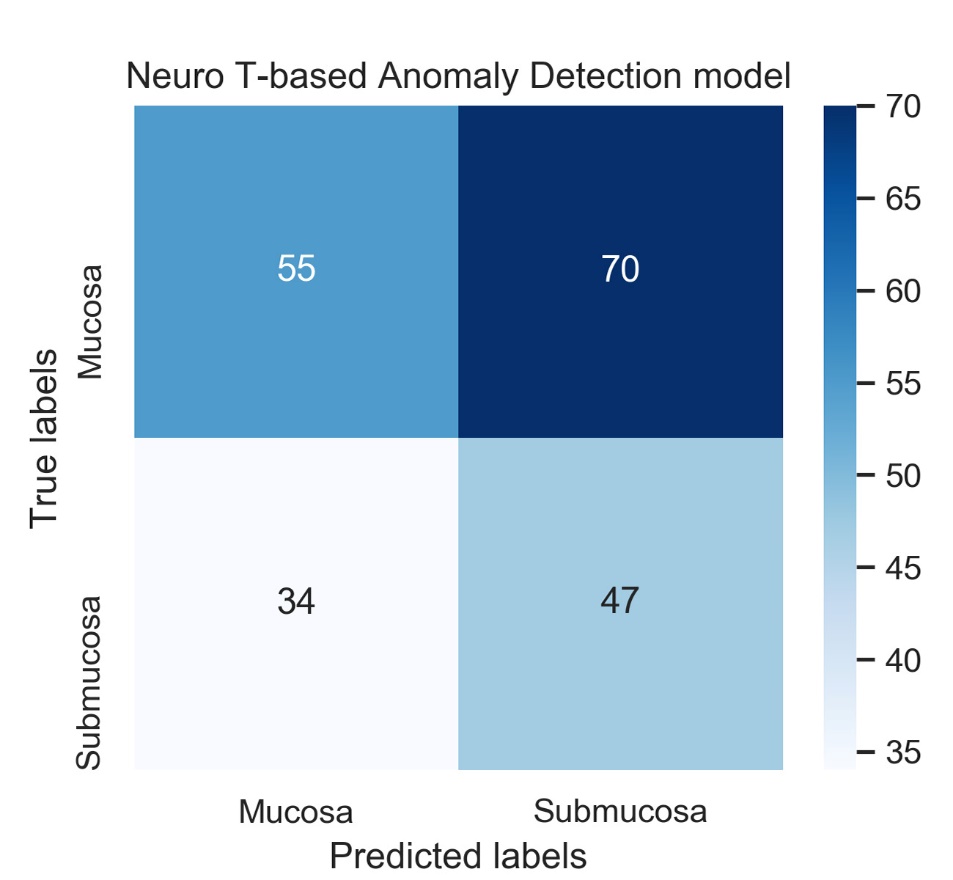


(D)


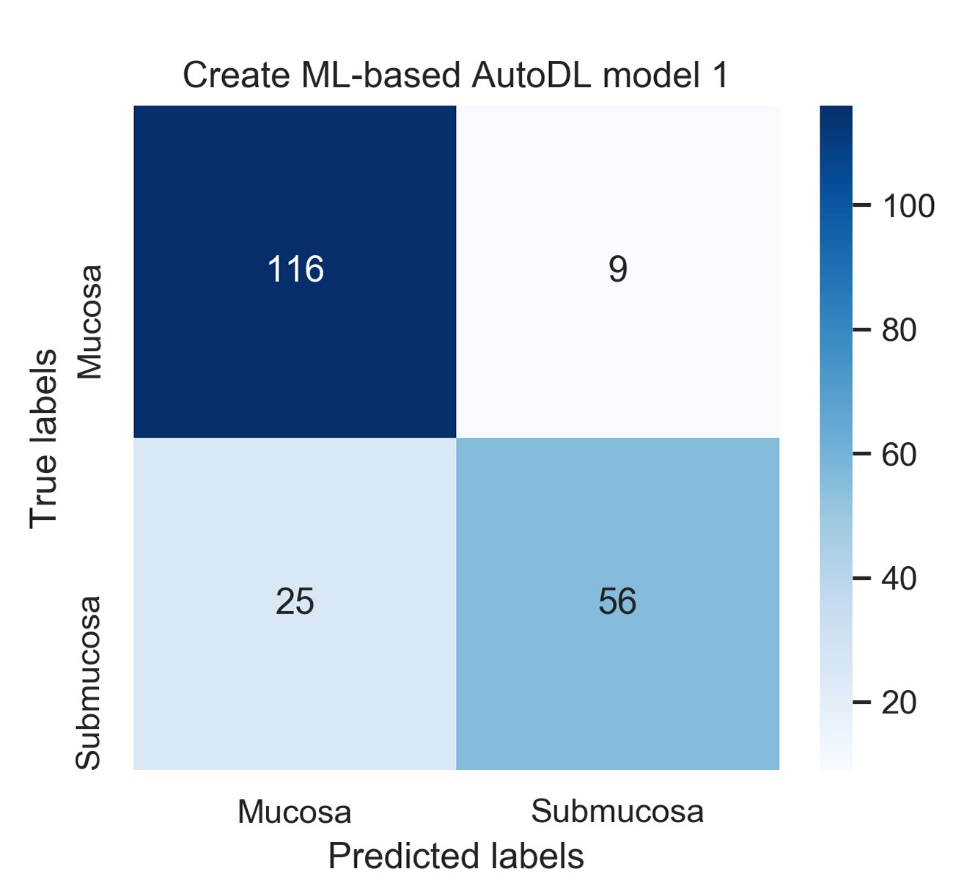


(E)


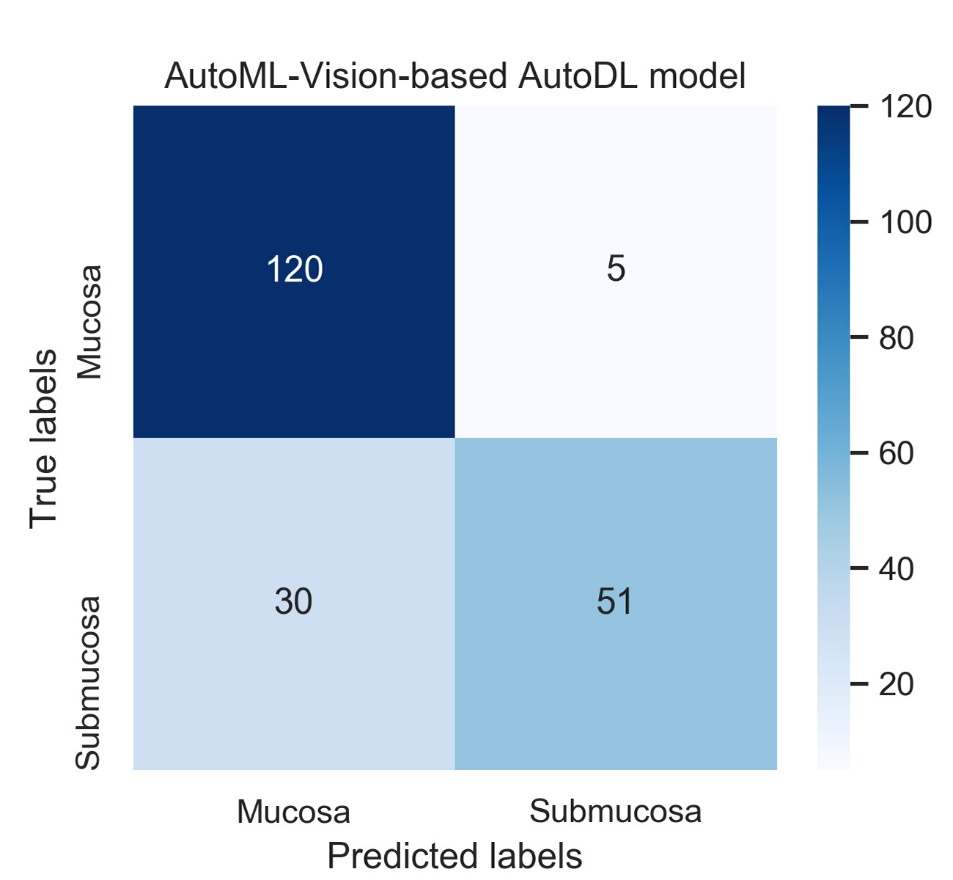

Supplement: Multimedia Appendix 2 [file jmir_v23i4e25167_app2.docx]
