# Supplementary material for: Use of Endoscopic Images in the Prediction of Submucosal Invasion of Gastric Neoplasms: Automated Deep Learning Model Development and Usability Study
Source: J Med Internet Res. 2021 Apr 15;23(4):e25167. doi: 10.2196/25167 (PMC8085753; doi:10.2196/25167)

**Multimedia Appendix** **3** Hypothetical clinical application of the automated deep-learning model for the determination of treatments based on the invasion depth of the lesion in an external-test.


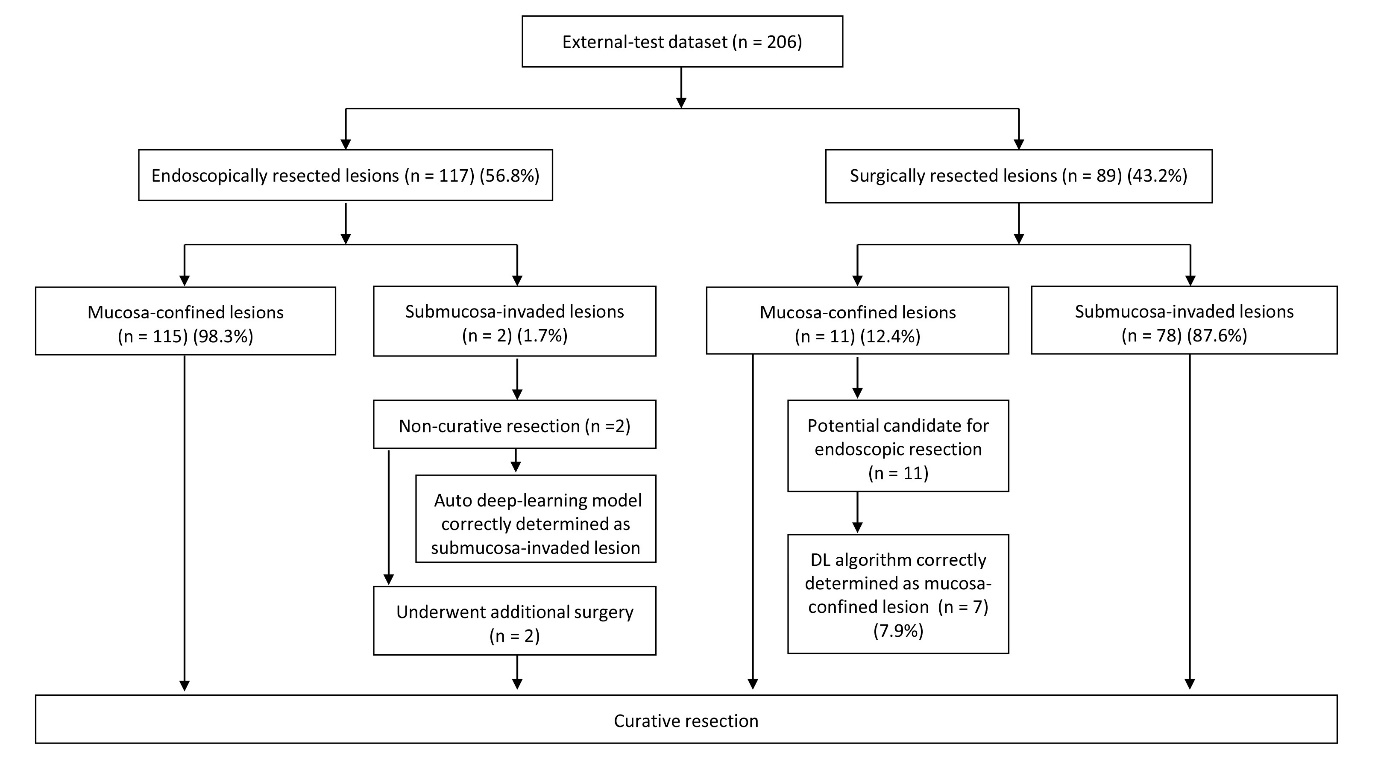

Supplement: Multimedia Appendix 3 [file jmir_v23i4e25167_app3.docx]
